# Unpacking the ‘15-Minute City’ via 6G, IoT, and Digital Twins: Towards a New Narrative for Increasing Urban Efficiency, Resilience, and Sustainability

**DOI:** 10.3390/s22041369

**Published:** 2022-02-10

**Authors:** Zaheer Allam, Simon Elias Bibri, David S. Jones, Didier Chabaud, Carlos Moreno

**Affiliations:** 1 Chaire Entrepreneuriat Territoire Innovation (ETI), IAE Paris—Sorbonne Business School, Université Paris 1 Panthéon-Sorbonne, 75013 Paris, France; chabaud.iae@univ-paris1.fr (D.C.); carlos.moreno@univ-paris1.fr (C.M.); 2 Live+Smart Research Laboratory, School of Architecture and Built Environment, Deakin University, Geelong, VIC 3220, Australia; 3 Department of Computer Science, Norwegian University of Science and Technology, Sem Saelands veie 9, NO-7491 Trondheim, Norway; simoe@ntnu.no; 4 Department of Architecture and Planning, Norwegian University of Science and Technology, Alfred Getz vei 3, Sentralbygg 1, 5th Floor, NO-7491 Trondheim, Norway; 5 Wadawurrung Traditional Owners Aboriginal Corporation, 86 Mercer Street, Geelong, VIC 3220, Australia; davidsjones2020@gmail.com; 6 Cities Research Institute, Griffith University, 170 Kessels Road, Nathan, QLD 4111, Australia

**Keywords:** smart cities, Internet of Things (IoT), sensors, 6G, wireless communications, resilience, sustainability, climate change, connectivity, data

## Abstract

The ‘15-minute city’ concept is emerging as a potent urban regeneration model in post-pandemic cities, offering new vantage points on liveability and urban health. While the concept is primarily geared towards rethinking urban morphologies, it can be furthered via the adoption of Smart Cities network technologies to provide tailored pathways to respond to contextualised challenges through the advent of data mining and processing to better inform urban decision-making processes. We argue that the ‘15-minute city’ concept can value-add from Smart City network technologies in particular through Digital Twins, Internet of Things (IoT), and 6G. The data gathered by these technologies, and processed via Machine Learning techniques, can unveil new patterns to understand the characteristics of urban fabrics. Collectively, those dimensions, unpacked to support the ‘15-minute city’ concept, can provide new opportunities to redefine agendas to better respond to economic and societal needs as well as align more closely with environmental commitments, including the United Nations’ Sustainable Development Goal 11 and the New Urban Agenda. This perspective paper presents new sets of opportunities for cities arguing that these new connectivities should be explored now so that appropriate protocols can be devised and so that urban agendas can be recalibrated to prepare for upcoming technology advances, opening new pathways for urban regeneration and resilience crafting.

## 1. Introduction

Cities across the globe have undergone notable transformations, especially following the different waves of the industrial revolutions witnessed since the 18th century. Underpinned by these waves, contemporary cities are now experiencing a transformation hinged on widespread technological integration, with diverse and city-specific outcomes being expected. Of this, the most notable objective and outcome being pursued by these cities is the increasing efficiency and performance in different urban frontiers [1]. Technological integration in different elements of cities is enveloped within the Smart City concept. Proponents of the concept envision an urban environment characterised by reduced human interventions as a result of automation of different urban elements in diverse geographical locations globally. However, automation, being one aspect of ‘smartness’, is dependent on the amount, quality, and type of data that different urban elements generate [2]. Thus, it is credible to argue that data are becoming a cornerstone of urban planning practice. Therefore, data collection, storage, analysis, and interpretation is critical, especially in helping to understand different urban dynamics and in scaffolding more informed decision making [3].

In addition to data, the smart city concept is further grounded upon the availability of other diverse and advanced technologies that not only allow for data collection, storage, and exploitation but also permit for technologies that help in implementing decisions and insights after data are synthesised, analysed, and interpreted. Such technologies include Artificial Intelligence (AI), Machine Learning (ML), Crowd Computing (CC), connectivity technologies such as 5G (including anticipated 6G), Robotics, and many others [4]. When compounded, the bulk of these technologies, plus different urban elements are seen to make smart cities and their market attractiveness very lucrative.

Currently, the smart cities ‘industry’ is valued at approximately USD 741.6 billion. With the attention on its sustained implementation amid the prevailing global pandemic challenges, this industry is expected to grow substantially to over USD 2.5 trillion by 2026 [5]. While there are other sources that estimate the market value of this industry differently (for example, Marekts and Markets [6] estimates it to be currently worth USD 457 billion and to grow to USD 873.7 billion by 2026), it is evident that regardless of different market valuations, the concept is promising and is expected to contribute to strategic urban planning trends globally. In particular, beyond the economic frontier, the smart city concept is expected to continue providing opportunities for increased liveability standards, increased sustainability prospects, and improved social dimensions amongst other unparralleled benefits [7,8,9].

While the smart cities concept is still gaining traction, the COVID-19 pandemic has prompted the emergence of a new urban planning concept—the ’15-minute city’. This city type focuses more on promoting social dimensions, urban proximity, and diversity via increasing use of technologies [10]. While the ’15-minute city’ concept will be described comprehensively in the next section, it is worth noting that the concept has only been described in the literature since 2016, with authors noting commonalities including social distancing, work-from-home concepts, and reduced travel movements, and the concept is increasingly becoming associated in tandem with the smart city concept in crafting and structuring more liveable and human scale cities [11]. However, this urban planning model promises to introduce new characteristics, such as proximity-based approaches to the planning of urban amenities, and urban restructuring, especially in relation to existing urban infrastructures and other elements, so that ultimately, there is a need to mediate these new aspects to human dimensions and values.

The 15-minute city concept, when unpackaged, both in the global north and south, is expected to transform urban areas and allow them to become more human-friendly, especially in the shadow of the post-pandemic ‘new normal’. Furthermore, this concept will prompt urban areas to better align for prospective future post-pandemic urban morphologies, especially with prospects that new concepts and technologies (e.g., metaverse) may have in prompting urban restructuring, greenfield policy reinvention, and regeneration generally. The contributions of this paper to the knowledge on 15-minute city concept include the following:

- Comprehensively showcases that the adoption of smart technologies can render more inclusive urban fabrics.
- Explores the adoption of the concept within the new realities (new normal) prompted by COVID-19.
- Explains in detail how emerging technologies such as the Digital Twins and the anticipated 6G technology will further accelerate the adoption and success of this new planning model.
- Highlights some possible obstacles that will need to be overcome to ensure that the anticipated benefits, especially on the social front, are realised.

Within this context, this paper seeks to explore the technological dimensions that are influencing the adoption and implementation of the 15-minute city concept in different global cities. This appraisal includes introducing the ’15-minute city’ concept in Section 2; Section 3 situates its relationship within Smart Cities practices and relevant techniques and technologies; Section 4 unpacks the concept’s technological dimensions; and Section 5 presents a discussion and conclusions.

## 2. The ‘15-Minute City’

The ‘15-minute city’ concept is a new urban planning model conceived in 2016 by Franco-Colombian scientist Carlos Moreno, a specialist in intelligent control of complex systems, who envisioned the need for urban environments to be people-centred [12,13]. Moreno acknowledges that he drew inspiration from Jane Jacobs’ writings [14]. His model gained prominence with its electoral advancement by Paris Mayor Anne Hidalgo within her “living smart city” initiative called the “Ville du quart d’heure”—the 15-minute city [15].

Moreno’s concept is that within an urban area, where human aspects such as socialisation, self-actualisation, cultural demand, and health, among others, the time required for people to access different nodes within the space is given precedence and priority during city planning. This policy empowers that the placement of essential urban amenities, infrastructures, and opportunities is deliberately actioned to facilitate enhanced accessibility. With policy implementation, it becomes possible for residents within given urban areas to comfortably walk or cycle to any given node within a city in a timeframe not exceeding 15 minute [16]. Thus, the demand for the use of automobiles to travel within the city is reduced, providing room for opportunities to create walkways and bicycle lanes that would have been otherwise suppressed in conventional urban planning models that prioritise vehicular flows’ efficiencies. Therefore, the 15-minute city concept seeks to bring a paradigm shift in the way urban planning has been previously practiced, shifting it from one focused upon vehicular flows, resulting in gridlocked cities, being a deterrent to the human societal endeavours and city liveability.

Moreno’s ‘15-minute city’ concept is inspired from ‘Chrono-urbanism’, where the aspect of time is believed to be a key factor to consider relative to space [10,13]. That is, the act of placing of different urban amenities and different elements needs to be guided by how much time it would take a walking or a cycling resident to move from one node to the next. In essence, even in urban areas endowed with maximum space, the proximity of different urban elements needs to be a critical consideration.

**Figure 1:**
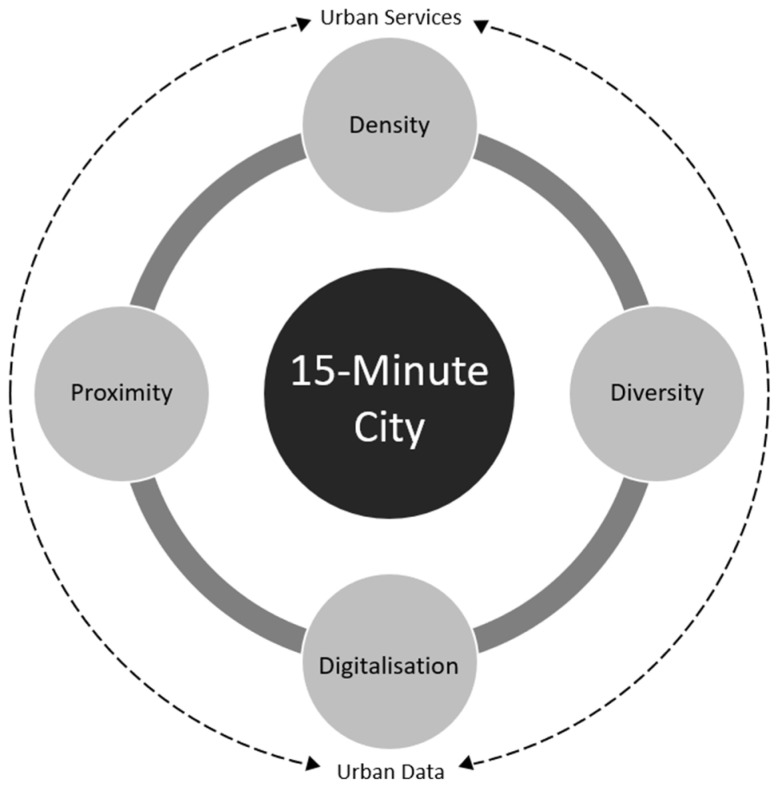
The 15-minute city framework as introduced by Moreno, Allam, Chabaud, Gall, and Pratlong [11].

Within this concept, it is possible to structure a number of nodes within a city, as long as all these observe the four key characteristics (as shown in Figure 1 below) that Moreno, Allam, Chabaud, Gall and Pratlong [11] argue are key in driving urban liveability. These include proximity, diversity, ubiquitousness, and density. In regard to diversity, the vision is to render urban areas accommodating of people from given backgrounds, thus promoting cultural vibrancy, while ensuring that there is diversity in terms of urban structures. That is, planners need to ensure that each of the urban structures, infrastructure, and elements could be used utilised for multiple purposes, hence allowing for their maximum utilisation [12]. For instance, in the case of neighbourhoods, there are opportunities for building urban structures such as car parks that would have capacities for multiple use. Such a move would ensure that there is maximum utility derived from buildings and urban public spaces. Therefore, there is the capacity to craft sufficient urban spaces for the creation of other critical amenities within the same neighbourhoods. Another example is the utilisation of school playing grounds for other purposes including parking, and recreation centres, especially external to school time whether on the surface, above, or below.

In terms of density, the 15-minute city concept espouses that cities should have an optimal number of residents. This assumption thereby ensures that it is possible to facilitate quality resource and service provision, without over-consumption or under-utilisation. In respect to the theme of this paper, the density dimension is critical in terms of data generation, which in turn helps not only with influencing how resources are utilised but also in feeding the virtual model of the city, thereby helping in rendering improved urban dimensions. The ubiquitousness principle advances the need for 15-minute cities’ requirements to be in large supply in all geographies, thereupon making them available for everyone and at an affordable cost. This aspect will greatly benefit from the deployment of the three technologies (IoT, Digital Twins, and 6G) being advanced in this paper. We argue that with these technologies, it will be possible for urban planners to contextualise and implement 15-minute city models. Additionally, through using the aforementioned technologies, it will be possible to customise each model to varying geographies to fully address place-relevant human dimensions. Thus, such technologies will help in fast tracking those customisations and implementation as is already being done in cities such as Paris, where the agenda is to reduce private cars with a target of 50%, while ensuring cycling-friendly environments through the creation of more bicycle lanes in the city. Another city implementing this concept is Bellevue through its *Environmental Stewardship Plan 2021–2025*.

## 3. The ’15-Minute City’ via the Smart City Network

One of the key dimensions of the 15-minute city is digitalisation. This entails the use of digital technologies to influence how the city functions and thus deliver services as well as provide value-producing opportunities in relation to various urban systems and domains. The latter relate to the overall landscape of the 15-minute city in terms of its underlying components, including ICT infrastructure, built infrastructure, green/blue infrastructure, transport infrastructure, energy infrastructure, economic infrastructure, and social infrastructure. Against the backdrop of this perspective paper, the focus is mainly on the ICT infrastructure and the built infrastructure given their particular relevance to the dimensions of the 15-minute city. The built infrastructure denotes the following:

“… the patterns of the physical objects in the city pertaining to the built-up areas as well as those areas planned for new development and redevelopment … The compact and ecological dimensions of urban design characterize most of the built infrastructure as regards its buildings, blocks, streets, open space, public space, green space, and essential infrastructure” [17].

The core design strategies shared by both the compact city and the eco-city are density, diversity, and proximity enabled by mixed land use—which are strategies that also characterise the 15-minute city. ICT infrastructure enables a 15-minute city to move to a data-driven form of urbanism by leveraging advanced data and information technologies to entirely transform its processes and practices—evaluating, analysing, re-engineering, and envisioning the way urban infrastructures and services can be designed, developed, managed, and planned in line with the vision of sustainability. ICT infrastructure digitally consists of those components that power the technology that pervades the fabric of the city (e.g., sensors, smart devices, systems, software programs, networks, data storage facilities, data processing platforms, cloud and fog computing, policies, and standards) and thus permeates urban life, providing support for the management of the city. To coordinate the many different components that comprise the digitalisation dimension of the 15-minute city requires a much stronger function of intelligence. This brings together what government and business have to offer in terms of engaging users of services and communities and providing hardware, software, and solutions enabling smartness, respectively. In this respect, among the issues deemed important are the ways in which ICT infrastructure can be integrated and coordinated, how the data can be analysed and harnessed, and how services can be delivered in a more efficient way. With respect to the latter, digital infrastructure is critical in remote areas in improving not only the efficiency of infrastructure networks but also their sustainability and services (e.g., energy, mobility, transport).

ICT infrastructure can be deployed within the 15-minute city’s own facilities (or cloud computing) in order to deliver solutions to different stakeholders with respect to services and applications. In this regard, ICT infrastructure should initiate innovative approaches to the use and integration of IoT, AI, AIoT, big data analytics, simulation models, and intelligent decision-support systems as part of urban computing to enable urban intelligence (e.g., enhancing mobility, reducing congestion, lowering energy use, reducing air pollution, improving planning, optimising governance, etc.). The purpose of this approach will be towards solving problems and issues related to the 15-minute city’s operational management and development planning. As related to urban computing, the efforts “… dedicated to connecting unobtrusive and ubiquitous sensing technologies, advanced data management and analytics models, and novel visualisation methods to structure intelligent urban computing systems for smart cities …” [18] can be utilised to develop innovative solutions in the form of applied urban intelligence for the management, planning, and governance of the 15-minute city.

Unsurprisingly, urban computing and intelligence is increasingly gaining momentum in academic circles and policy debates as a policy agenda for integrated advanced technologies and their novel smart applications for tackling many of the contemporary complex problems and challenges associated with urbanisation and sustainability.

[As] “… a process of acquisition, integration, and analysis of big and heterogeneous data generated by a diversity of sources in urban spaces, such as sensors, devices, vehicles, buildings and humans, to tackle the major issues that cities face …” [urban computing] “… create win–win–win solutions that improve urban environment, human life quality, and city operation systems … [and] also helps us understand the nature of urban phenomena [and urban dynamics] and even predict the future of cities …” [19].

**Figure 2:**
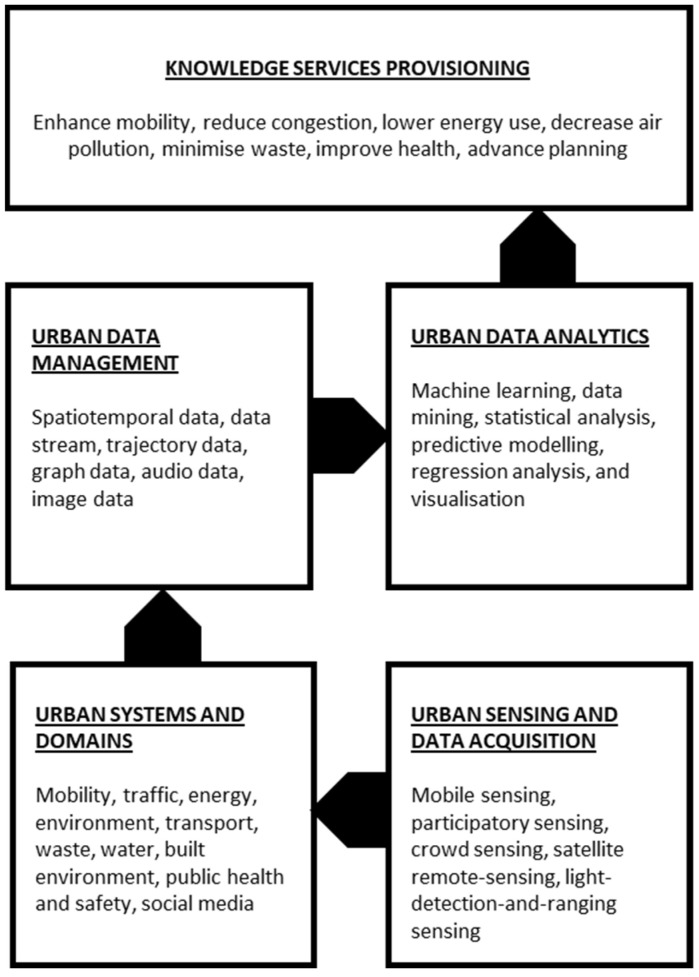
General architecture for urban computing and intelligence based on big data analytics. Illustration by authors.

As an integrated and holistic approach, urban computing and intelligence makes it possible to generate well-informed decisions concerning a wide range of city services and operations, and it can also enable feedback loops between urban environments, human activities, and physical movements [20]. The analytical process in this approach enables the creation of knowledge services required for enhancing decision making based on the design of the components and their relationships, as illustrated in Figure 2.

With the escalating rate of urbanisation and mounting challenges of sustainability, it has become of crucial importance to develop a new urban fabric that can deal effectively with urban development in regard to its dimensions—namely land use change, population increase, cultural change, and economic growth, through such design strategies as compactness, density, diversity, and mixed land use. In this context, an urban fabric refers to

“… the physical characteristics of urban areas in terms of components, buildings, spatial patterns, scales, streetscapes, infrastructure, networks, and functions, as well as socio-cultural, ecological, economic, and organizational structures …” [21].

This also involves making the best use of the digital and informational assets to ensure that the city is sustainable in its approaches to integrating new technologies and their novel applications with compact design strategies. This requires implementing an advanced form of urban computing for monitoring, measuring, analysing, evaluating, designing, and planning urban systems, thereby enabling many functions of urban intelligence for the purpose of improving the sustainability, efficiency, resilience, and life quality in the 15-minute city.

Within this context, IoT has recently become the predominant paradigm of urban computing and intelligence, shifting from a vision of ICT of ubiquitous computing towards one of a deployable paradigm. This shift heralds a new wave of city analytics whose basic ingredient is big data analytics [22,23,24], which is fostered by the proliferation and widespread diffusion of wireless communication technologies on a hard-to-imagine scale. This is manifested mainly in the quantity and scale of Wi-Fi hotspots covering many urban areas to form a dense multi-faceted IoT network necessitating a large number of sensors exhaustively deployed across the city in order to enhance their communication capabilities and data transfer processes.

Given the wide array of its network in urban areas, via smart city networks, IoT has been extensively installed and used in cities without many engineering obstacles as regards resources, buildings, and infrastructures. IoT infrastructure, involving a myriad of devices seamlessly connected for information exchange, is used to collect vast troves of data to aid in enhancing and optimising urban operations, functions, designs, and policies in relation to various urban domains. IoT when coupled with the data deluge flowing through its multiple networks of sensors plays a key role in the development and implementation of the 15-minute city as a new concept, serving as a technological backbone to the city’s attempts to address its goals of sustainability with respect to its underlying dimensions. As an unprecedented planning effort, the 15-minute city initiative is a response to the deconcentration of land use, and as such, it emphasises density, diversity, and mixed land use as key strategies for ensuring liveability, vitality, affordability, energy conservation, and environmental quality. This emerging approach to urban development seeks to deliver more efficient land use, build a resilient and adaptable urban community, lower per capita rates of energy usage and per capita infrastructure provision, and thereby reduce pollution thanks to density and proximity.

Further, IoT infrastructure is necessary to fulfil the needs and visions of the 15-minute city as a smart sustainable approach to urban development. IoT is seen as key to enabling both the smart city [1] and sustainable city infrastructure [25], as it provides a flexible infrastructure that is of crucial importance to deal with the myriad of interconnected devices. It is important for the 15-minute city to have IoT infrastructure in place, where end-device connectivity is monitored, communication reliability is assured, and its sub-systems are intelligent enough to communicate and exchange information with one another while forming a large-scale digital system with widely deployed devices to enable services [26,27,28] associated with sustainable urban living. A successful implementation of IoT in the 15-minute city means

“… supporting the complexity of different sensors and their networks set up in urban environments as well as simplifying the composition of interoperable services and applications. Sensor–enabled smart objects are regarded as the essential feature of the interconnected infrastructures of the future” [29].

IoT is an advanced form of ICT of ubiquitous computing. It includes an array of ICT architectures that are fundamentally aimed at describing and providing the relevant infrastructure that underlie the functioning of the digital ecosystem of the city—urban computing and intelligence—within both smart cities [30] and sustainable cities [29]. Thus, ICT architecture denotes a framework for the design of the components and their relationships, functioning as a kind of a roadmap to a city’s ICT aspects: for example, what needs to be done to respond to the city’s digital needs. ICT infrastructure, in contrast, includes the assets themselves that are used in the city, such as hardware, software, networks, computers, towers, servers, and so forth. Accordingly, the architectural design of ICT determines the variety and number of technologies that can be included in the ICT infrastructure. In essence, a digital ecosystem is built on an infrastructure that has a particular architecture. Therefore, it is impossible to use a particular architecture or infrastructure as a blueprint for all possible implementations in real-world settings. In other words, there is no single consensus on architecture for ICT or infrastructure for ICT that can be agreed upon universally. Different cities have different architectures and different infrastructures, such as planning-based architecture, governance-based architecture, operations-based architecture, healthcare-based architecture, and smart home-based architecture and others.

Bibri and Krogstie [29] offer a detailed review of the key technological and computational components of IoT, including its relationship with big data technology and analytics, sensors and things, big data analytics as a holistic digital system, the core enabling technologies of big data ecosystem, big data analytics solutions, ICT architecture, and IoT infrastructure. Nevertheless, as an advanced approach to ICT design, IoT architecture tends to converge on the number of layers with regard to the design of the components that make up a technological system and their relationships. This still depends on the application domain [24,31,32,33,34]. Sometimes, the architectural layers are combined depending on the complexity of the application domain while using different, and sometimes overlapping, labels, such as the physical layer, perception layer, information source layer, middleware layer, network layer, technology layer, application layer, service layer, and domain layer. In the context of the 15-minute city, the four layers of IoT architecture include the following:

(1) Physical/Perception Layer: Urban sensing and data acquisition;
(2) Network/Transmission Layer: Data transfer and communication;
(3) Middleware/Technology Layer: Data hosting, management, processing, and analysis;
(4) Application/Service Layer: Service provisioning specific to urban domains.

### 3.1. Physical/Perception Layer

This layer works with various types of sensors to generate and collect the data from different sources from across urban systems and domains based on sensor-centric and human-centric sensing mode. A sensor is a device that converts signals from one energy domain to an electrical domain.

Urban sensing includes mobile sensing, participatory sensing, crowd sensing, satellite remote-sensing, and light-detection-and-ranging (LIDAR) sensing. For example, these pertain to mobility, traffic, energy, environment, road networks, transport systems, and built objects. Accordingly, the billions of connected devices forming the IoT infrastructure across a city are equipped with sensors to collect data about the way they are used as well as about the environment surrounding them, with built-in wireless connectivity and communication capabilities enabling them to exchange the generated data. One of the key features of sensors, once deployed, is their ability to interpret the data received from the surrounding environment and generate an output. Sensors measure physical input and send signals to the processor, converting it into data that can be interpreted by a machine or a human. With respect to the former, sensors send their readings to a backend system with humans being left out of the loop. In essence, sensors have the ability to convert data obtained from the outside world into a format that can be preprocessed and further processed and analysed. Sensors are the core enabling technology of IoT, providing an automated approach to urban data generation and thus serve as the main source for big data management and analytics as a form of large-scale computation through middleware. However, among the challenges of urban sensing are resource deployment, implicit and noisy data, skewed sample data, and data sparsity and missing data [35,36,37].

Raw data are generated about a city in terms of its urban environments, human activities, and physical movements by means of a broad network of sensors spread across the city, including radio frequency identification (RFID) tags, near-field communication (NFC), accelerometers, surveillance cameras, LIDAR, transponders, smart metres, global positioning system (GPS), transduction loops, smartphones, and a number of other digital platforms generating ranges of real-time data. In terms of sensors associated with human mobility and activity, sensors leverage humans as data agents to investigate urban phenomena and dynamics during their movements in urban areas for the purposes of solving and servicing urban problems collectively.

### 3.2. Network/Transmission Layer

This layer acts as a bridge between the physical layer and the application layer through a middleware layer. It includes a dispersed network as a set of technologies and solutions that allows the transmission of the acquired data for further processing and analysis. As such, it carries and transfers the data collected from the physical objects through sensors by means of multiple wireless networking technologies that provide continuous data regarding the physical and social forms of the city, including Wi-Fi, Bluetooth, satellite, cellular (4G/5G/6G), Local Area Network (LAN), Low Power Wide Area Networks (LPWANs), Zigbee (a low-cost, low-power, wireless mesh network standard targeted at battery-powered devices in wireless control and monitoring applications), and other mesh protocols. While IT devices traditionally are connected to a central access point, satellite, or cell tower somewhere, relying upon expensive hardware infrastructure, mesh networking devices connect directly to each other. Thus, IoT is predicated upon making it possible for about anything to be wirelessly connected and to communicate data over a multiplicity of networks.

### 3.3. The Middleware/Technology Layer

This layer provides a connectivity layer for the physical layer and for the application layer. As such, it serves as an interface between the varied components of the IoT architecture, making communication and possible among different, often complex, and already existing elements and programs. It contains the main framework for organising and centralising the data collected from the sensor network. One of the key functions of middleware, which operates on cloud computing, is handling the distribution, heterogeneity, interoperability, dynamicity, and scalability of computing resources and systems related to the logic of IoT applications. Middleware is at the core of IoT as a pervasive computing environment, distributing applications across urban domains. It empowers distributed processing for information fusion from multiple components of the physical layer [38]. It is the logic glue with respect to the functionality of distributed applications by connecting and coordinating many components of the IoT as a complex distributed computing system. This involves a variety of the heterogeneous hardware and software elements that are highly interoperable and dynamic, involving a myriad of embedded devices and information processing units required for scaffolding the IoT environment and its proper functioning.

Middleware is necessary for bridging the gap between the massively embedded and networked devices and systems elevating IoT as a form of urban computing and intelligence as it allows multiple processes to run on various sensors, computers, and networks to link up, interact, and communicate to support and maintain the operation of IoT applications. The scope is the ability of an ensemble of devices, smartphones, computers, databases, data warehouses, application integration methods, application servers, application networks, web servers, content management systems, messaging systems, routing, and message transformation to cooperate, interconnect, and communicate seamlessly across disparate networks that create the IoT environment rather than their pervasiveness and extensiveness. Furthermore, middleware supports and deploys numerous applications networks across large geographical areas that are created by sensor networks, network–monitoring systems, and dynamic Web, and that collaborate with, or leverage services from, other disparate systematically tied applications using integration approaches [24].

Middleware is a multi-layered architecture in itself, which comprises four distinct sub-layers, namely [39]:

1. Host–infrastructure middleware or infrastructure and communication;
2. Semantic services and agents or distribution middleware;
3. Common middleware services or services for software environment;
4. Intelligence or domain-specific services related to application action coordination.

Considering the last two sub-layers, the middleware layer also provides processing and analytics procedures to obtain the meaningful information or extract the useful knowledge for numerous applications [23]. This pertains to both urban data management using cloud computing platforms, indexing structures, and retrieval algorithms in regard to spatio-temporal data [19], as well as urban data analytics in terms of adopting machine-learning and data-mining algorithms and models to extract useful knowledge from data across different urban domains using such supervised and unsupervised techniques such as classification, clustering, regression, causal modelling, predictive modelling, and profiling. The process of urban data analytics also fuses the knowledge from multiple disparate datasets across domains [40], using such methods as deep learning-based [35], multi-view-based, transfer learning-based data fusion, similarity-based, and probabilistic dependency-based [19].

### 3.4. The Application/Service Layer

This layer involves a varied set of applications that use the meaningful information obtained from the physical and middleware layers. Accordingly, this layer provides a wide range of knowledge services for a city in the form of applied intelligence functions. The categories of services provided in this regard are based on the common types of big data analytics, namely diagnostic, descriptive, predictive, and prescriptive, and the domain for which these services are created. Furthermore, this layer offers interfaces that allow urban domain systems to call the knowledge from an IoT application through a city’s own facilities (or cloud computing platforms), where the knowledge extracted from data must be integrated into decision-support processes in existing urban domain systems to inform their decision making. This includes visual analytics for model exploration, simulation and prediction methods, and distributed data mining or knowledge discovery strategies. These processes in turn entail distributed data mining and network analytics, extracted models underpinning management and evaluation, and model construction for making assumptions and powerful predictions enabled by mining through AI to improve decision-making processes. In particular, it is of vital importance to enable interactive visual analytics [41], which “… combine human wisdom with machine intelligence by keeping domain experts in a learning loop” [19]. This layer also relates to urban dashboards and smart boards with visualisation in relation to management system control, automated response systems, and other types of applications. It offers a connectivity of an extensive and transversal resources to multiple users and consumers enabling the adoption of data-driven smart solutions.

Accordingly, 15-minute city architecture is organised into four layers: (1) data generation, (2) data transmission, (3) data management and analytics, and (4) smart services for applications. The services supporting the 15-minute city architecture benefit from the analytical outcome of urban data. Thus, they are provided based upon sensor-based data that is abundant, holistic, dynamic, fine-grained, relational, resolute, and actionable thanks to the intensification of datafication of contemporary cities, allowing for real-time analysis and innovative and adaptive forms of city management and planning. Concerning the latter, IoT architecture for the 15-minute city involves a whole collection of data-driven smart solutions for various urban systems and domains. Such solutions can be adopted by city management agencies and city planning centres to serve various stakeholders by improving sustainability, optimising efficiency, strengthening resilience, and enhancing life quality.

## 4. Unpacking the ‘15-Minute City’ via Tech-Centric Approaches

### 4.1. The ‘15-Minute City’ and Digital Twins

Digital twins is an emerging technology that allows for the virtual representation of the physical objects, processes, and services in the virtual environment [4]. With this technology, it has become possible to create replicas that different stakeholders are able to interact within the virtual world, just as in the real world. This then makes it possible to virtualise, analyse, simulate, test, and map different aspects and scenarios in the virtual world to help in making informed decisions, including predictions and modelling that ultimately influence situations in the physical world [42]. In this context, the backbone of the Digital Twins (DT) technology, as argued by Dontha [43], is the availability of real-time, massive, and quality data of a given object, or process that is collected from multiple sources and later fed into the virtual model; hence, it allows us to understand the real situation in the physical world. With data, Qi and Tao [44] note that it is becoming possible for different players in the global spheres to predict future scenarios as well optimise performances.

For cities, especially those that have already embraced some aspects of the smart city concept, they now have the capacity and means to not only generate enough data but also have systems to collect and store the data. Accordingly, DT technology is expected to play a critical role in urban regeneration and its implementation [4]. Indeed, DT could help in the introduction and fashioning of improved regeneration models, as DT technology would allow for the simulation and testing of different scenarios. DT technology would in particular benefit from dynamic models of decision making that allow for participatory planning models that in turn make it possible for data to be collected even from different communities, especially in regard to their expectations and aspirations [45]. With such data, it would then be possible to simulate the impacts of those expectations on the urban areas in case they are factored in, in the regeneration models, including in the adoption of the ‘15-minute city’ concept. Ultimately, it becomes possible for the urban planners and designers to incorporate urban dwellers’ expectations, which guarantees optimal impacts and also allows for maximum acceptance and increases ‘ownership’ of the regeneration model. For instance, in the pursuit of the ‘15-minute city’ concept, the integration of DT technology in the urban planning approaches would allow residents to visualise, and where possible interact virtually, with the different anticipated scenarios and outcomes. Such opportunities would allow them to participate and propose the elements and components that they would wish to be included in their proposed urban regeneration model. However, on a negative tone, the integration of DT technology in the 15-minute city concept may prompt a number of disadvantages. For instance, this will render higher initial costs of implementation of the planning model, noting that this technology is also equally expensive. Furthermore, due to the financial needs to establish relevant infrastructures for DT technology, some cities may take longer to achieve as already, most cities do not have the capacities to finance such projects from their public budgets and have to rely on PPP programs and external loans. The DT technology is also in its infancy stage and will take time before it matures complete to be successfully implemented in the 15-minute cities.

### 4.2. The ‘15-Minute City’ and the Internet of Things (IoT) Network

The ‘15-minute city’ concept, as advanced by its proponents, may overly concentrate on the human-centric agendas and therefore, conceivably, could work sufficiently without the need for digital solutions. However, there is evidence that cities that have deployed elements of technology are able to improve aspects including quality of life for its residents by 10% to 30% [46], and they are also far better placed to achieve global accords and policy expectations including the United Nations’ (UN) Sustainable Development Goals (especially Goal 11 that seeks to “Make cities and human settlements inclusive, safe, resilient and sustainable”) [47] and the Glasgow Climate Pact emanating from the 26th UN Climate Change Conference (COP26) [48].

For the ‘15-minute city’ concept, while the vision is to render neighbourhoods that are compact and diverse, if not all services and amenities available are within reach, the integration of IoT networks and devices would further underpin the benefits being sought. On this, Turner and Townsend [49] note that ‘15-minute cities’ would benefit from the use of smart technologies in areas such as pollution warning, controlling, managing, and implementing local traffic and parking policies, and in providing real-time information on issues such as weather and emissions.

The massive data generated from different smart IoT devices in different nodes within ‘15-minute cities’ would further help in the adoption of modern solutions including smart lighting, especially for street lighting projects, smart parking, traffic light coordination, bike sharing, and others. With the data, for instance, it would be possible to effectively estimate local energy consumption rates enabling the adoption of economic and energy-efficient lighting programs that meet place-specific needs without unnecessary generic consumption [50]. Similar benefits would be derived in respect to the demand and consumption of other resources and the utilisation of available public spaces and infrastructures, thereby allowing for informed decision making in the provision of services. On this, one of the positive aspects of the ‘15-minute city’ concept is the prospect of addressing car dependency [51], by reducing the number of vehicles in cities through the provision of bicycle and walk-friendly environments for urban dwellers. Such can equally be rolled out in monitoring and measuring the health and oxygen generating of street trees and park trees, and their corresponding capacity in hosting animal and avian species offering new avenues to monitor and measure the biodiversity (and biophilic [52]) ecological health of a city [53]. With the adoption of smart technologies, Woods [50] showcases that such objectives can be achieved even quicker, as the availability of data could help identify priority areas as well as be used to help convince local residents of the benefits they would derive from adopting the new planning methods. However, to derive those benefits, a number of limitations associated with IoT and its applications will need to be addressed. First, the issue of data quality, data quantity, and privacy is critical. For instance, the case of privacy has been reported to prompt apprehension on the part of residents to embrace and participate in projects, and such predicaments could befall the 15-minute city concept if there are pitfalls regarding the guarantee for privacy and security. IoT technologies are also expensive; hence, they will require urban managers to commit extra financial resources, which might, in some scenarios, lead to plunging cities into increasing debt margins where the projects are already credit financed.

### 4.3. The ’15-Minute City’, 6G, and the Data-Driven Cities

As the number of IoT networks and devices continue to increase and become more advanced, we argue that there will be an increased demand for wireless connectivity and speed to facilitate real-time and fast connections [54]. However, although 5G networks have the capacity to help meet our current demands for connectivity, they are still not widespread, and most cities are only covered by the 4G networks, which are limited in their capacity to handle the demand for huge data transfers, real-time recordings, and other connectivity needs [55]. For 5G, Barakat et al. [56] argue that substantial resources including financing will need to be availed to ensure sufficient infrastructures are installed to facilitate the increasing demand emanating from both subscribers and the new devices and innovations that are powered by this technology. For subscribers, it is noted that by the first quarter of 2021, the numbers of those already subscribed had increased by 41%, and more will join following the realities of COVID-19 [57]. This means that in the near future, 5G technology will not be sufficient to support all the connection needs, especially in cities where the number of IoT devices are projected to increase to over 25.4 billion devices by 2030 from the current 8.74 billion devices available globally [58].

Then, the 6th generation (6G) of wireless communication will be inevitable, as the large number of smart IoT devices and networks and the subsequent increase in data will require a wireless technology that can facilitate quicker and real-time transfers from the point of generation to the relevant networks [4]. According to Nguyen et al. [59], 6G technology will bring to life the prospects of full intelligence and automations of systems. 6G technology will help actualise concepts such as the smart city, and in this case, it will be instrumental in helping realise the ‘15-minute city’ concept, which will also ride on the power of technology. In particular, 6G will allow for more user participation and efficiency in the correction and transfer of real-time data to the central networks for analysis and decision making. 6G will further allow for the emergence of new technologies such as the anticipated metaverse (a combination of multiple elements of technology, including virtual reality, augmented reality, and video where users “live” within a digital universe) that have prospects to help actualise the ‘15-minute city’, especially due to reduction of the need to travel, through its emphasis on remote working, and increase social aspects.

However, the 6G technology might take time before the prerequisite infrastructures are put in place before the technology undergoes the relevant piloting and testing, prior to eventually being launched. Whereas the 15-minute city concept is also still very new, it might not immediately benefit from the prospects of 6G as expressed here; thus, it will have to content with available technologies and find alternative ways of overcoming shortcomings that would otherwise be eased by the 6G technology.

## 5. Discussions and Conclusions

The smart city concept emerged and gained popularity in parallel to the equally exponential growth and increase in the use of advanced technologies riding on internet connections that came about with the fourth industrial revolution wave [60]. In particular, the technologies that emerged and continue to advance to date have initiated a new wave of data generation and also correction, and as such, they have been viewed to increase the prospect of efficiency in many frontiers such as decision making, improving performance, and allowing for real-time predictions, among others [1]. In urban areas as explained above, data mining prospects are seen to be propitiously play critical roles in shaping discourses and trends in topics including climate change, biodiversity health, cultural heritage conservation, traffic management, First Nations’ Country Plans [61], adoption of alternative energy such as rooftop solar energy, etc. However, while those benefits amass and will continue to emerge, it is worthwhile to note that the smart city concept attracts notable and genuine criticism from different quarters, especially in regard to the collection, storage, analysis, management, and control of data [62] enveloping also human privacy concerns echoing Orwell’s dystopian social science fiction novel and cautionary tale [63]. Such criticisms, especially associated with handling the urban data by the private entities, are well placed, particularly in view of privacy and security of personal data, and also the fear of monetisation thereof that is synonymous with most profit-oriented private entities [64].

Empowering the local government is particularly important as the ‘15-minute city’ concept promises to enhance people-centric dimensions and thereby is expected to generate large private datasets that would need high levels of privacy and security. With the assurance of data safety, it would be possible to convince urban residents to embrace the concept, enabling wider opportunities for urban managers to pursue the project, including adopting the smart urban technologies such as the DT, IoT, and the anticipated 6G technology.

From the literature above, it has been expressed how the DT technology holds unmatched potentials in helping actualise the ‘15-minute city’ concept. In particular, the ability to create a virtual replica of the city model will be critical, noting that the ‘15-minute city’ concept is an emerging technology that may face objections from people who do not understand how it may pan out. In a similar way, the DT technology has had successes in other sectors including manufacturing, the transport industry, and others, especially in providing opportunities for simulations and predictions [65]; it would be expected that DT could help actualise the ‘15-minute city’ concept and its implementation. As noted in the literature, this will be influenced by the availability of real-time data that is also quality and in emanating from different urban fabrics to ensure it allows for a real replica of the ‘15-minute city’ concept. Areas that would gain from this would include sustainability, resilience and quality of life, and the ability to predict and monitor urban health dimensions, such as air quality. These topics are increasingly the subject of new research activities leading to the emergence of varying air monitoring tools [66]. Such will be facilitated by the availability of IoT networks and devices that, as Liu [67] notes, will continue to increase, and new ones will emerge as more demand increases. Therefore, it will be safe to argue here that the prospect of the ‘15-minute city’ concept will not only benefit from existing IoT networks devices but will also play a critical role in influencing the emergence of new ones that help in its actualisation. It is possible that the increase in IoT devices, especially in advanced forms, will pose some connection challenges, and this then justifies the need for the 6G technology explained above. However, it is worth noting that while 6G technology is still in the pipeline, the early piloting of the ‘15-minute city’ concept can still benefit from the 5G technology, but the launch of 6G will revolutionise this concept, especially in view of the speed of transfer of data and increased capacities for communication between different smart devices installed.

The increased connectivity of devices, and the data gathered and processed, can further aid in automating urban dimensions [2,68,69], which can lead from ‘automation’ to ‘autonomy’ [70,71]. Of interest to this concept would be the need to replicate those automated features digitally for testing in varying scenarios [72], supporting the need for Digital Twins and the concept of ‘City Brains’. This convergence of AI-driven data processing and simulation can further aid in the better planning and implementation of the 15-minute city concept, leading to the contextual solutions, supporting the identity and character of neighbourhoods.

The need to align the ‘15-minute city’ concept with smart city networks would not need to be overemphasised. This is because it is already evident from the realities prompted by the global COVID-19 pandemic that data-driven cities hold the future for the urban areas. However, data alone are not enough. It will need to be exploited to help align urban areas with people-centric dimensions, which have not been sufficiently explored in existing smart cities [63]. With this element catered for, the ‘15-minute city’ concept is poised to be a successful urban planning model, with human scale elements sufficiently provided. However, this success will depend on how well this concept will be aligned with existing and emerging ‘smart’ technologies. Therefore, it could be safe to argue that the ‘15-minute city’ concept is emerging as a by-product, or an evolution, of the smart city narrative, and going into the future, it might become even more prominent and widely embraced within smart city models.

While this paper has anticipated some of the benefits that cities would accrue from a widespread acceptance and implementation of the 15-minute city concept, a follow-up study will be necessary to evaluate how the concept is taking shape. In particular, this will be relevant as some of the technologies appraised in this article are still very new, or some such as the 6G are still in the pipeline, and it will be prudent to report how those will eventually influence the 15-minute city model once they are deemed mature enough.
